# O039: Patient participation and performance feedback to improve hand hygiene adherence in the context of established multimodal hand hygiene promotion: initial results from a mixed-methods, cluster randomised trial

**DOI:** 10.1186/2047-2994-2-S1-O39

**Published:** 2013-06-20

**Authors:** AJ Stewardson, A Gayet-Ageron, S Touveneau, L Clack, M Schindler, W Zingg, M Bourrier, D Pittet, H Sax

**Affiliations:** 1 The Univ. of Geneva Hosp. and Fac. of Medicine, Geneva, Switzerland; 2 Univ. and University Hosp. of Zurich, Zurich, Switzerland; 3 Univ. of Geneva, Geneva, Switzerland; 4 University of Geneva Hospitals and Faculty of Medicine, Geneva, Switzerland

## Introduction

Hand hygiene (HH) compliance amongst healthcare workers is widely recognised as a key intervention in infection control. Given HH compliance remains sub-optimal despite standard multimodal promotion, there is an urgent need for evidence regarding the effectiveness of novel interventions.

## Objectives

To investigate the impact of optimised performance feedback (PF) and patient participation (PP) on HH compliance in the setting of a well-established multimodal HH promotion program.

## Methods

Single-centre, cluster-randomised controlled trial. After a 15-month baseline phase from April 2009, 66 wards were allocated by stratified randomisation to one of three arms during a 24-month intervention phase: control; PF; or PF+PP. Multimodal promotion continued in all three arms. PF was provided via cards, posters and emails. PP involved a partnership whereby healthcare workers and patients agree to remind each other to perform HH. The primary outcome was HH performance measured using the WHO ‘My 5 Moments’ methodology and analysed using a mixed effect logistic regression model. Qualitative data was gathered by focus groups and interviews with healthcare workers.

## Results

Twelve observers recorded 12,627 HH opportunities during 1,358 sessions. HH compliance was similar between arms at baseline and increased in all three arms during the intervention phase (P=0.04): 65% to 73%, odds ratio 1.36 (CI95% 1.17-1.59); 64% to 74%, OR 1.59 (1.39-1.81); and 64% to 76%, OR 1.77 (1.54-2.04), respectively, in control, PF and PF+PP arms. Only PP+FB showed a significant effect on HH compliance in our trial (OR 1.33, P=0.04), with PF alone not sufficient (OR 1.17, P=0.25). Qualitative data showed that acceptance and implementation of PP was gradual, variable and primarily dependent on ward leadership. Exclusion from intervention arms motivated control wards to improve HH performance independently.

## Conclusion

PP with PF may offer a means of improving HH compliance beyond standard multimodal promotion.

## Disclosure of interest

None declared.

